# Comparative assessment of outcomes of smoking cessation therapies and role of free medications in successful long-term abstinence

**DOI:** 10.18332/tid/136422

**Published:** 2021-06-15

**Authors:** Bengu Saylan, Seyma Baslilar, Zafer Kartaloglu

**Affiliations:** 1 Department of Chest Diseases, Sultan 2. Abdulhamid Han Sample Training and Research Hospital, Istanbul, Turkey; 2 Department of Chest Diseases, Umraniye Training and Research Hospital, Istanbul, Turkey

**Keywords:** smoking cessation, varenicline, bupropion, nicotine replacement therapy, psychosocial support

## Abstract

**INTRODUCTION:**

Long-term outcomes of smoking cessation treatments are crucial to optimize standards of cessation services, which are known to prevent excess morbidity and mortality. This study aimed to evaluate long-term outcomes of a smoking cessation program, to compare the success rates of interventions, to assess relapse rates after quitting, and to determine the duration until relapse.

**METHODS:**

Patients admitted for smoking cessation between 2010–2018 were contacted to evaluate short- and long-term treatment outcomes. The patients were asked whether they were currently smoking, and whether they quit after smoking cessation treatment and the duration of abstinence.

**RESULTS:**

The study included 579 patients (341 males) with a mean age of 50±12 years. The median time from the date of visit to the smoking cessation clinic to analysis was 5 years (range: 2–10). Of the patient, 436 used medications, including varenicline, bupropion, and nicotine replacement therapy (NRT). The overall quit rate was 31.8% by the primary intervention (varenicline: 45.5%, bupropion: 38.2%, NRT: 33%, psychosocial support: 4.2%), and quit rate was similar in the intervention groups (p=0.073). In the long-term, the quit rates were 19.6, 22.5, 25.9, and 21.7%, respectively (p=0.405). About 9% of the patients failed to quit smoking initially but succeeded for a while after the first intervention at the cessation clinic. The relapse rate after initial cessation was 19%. The longest period of abstinence was in patients using NRT (14±17 months), followed by the patients using varenicline (9.5±12.7 months) and bupropion (8.2±14.8 months).

**CONCLUSIONS:**

Both short- and long-term quit rates with varenicline, bupropion, and NRT, were similar. The long-term quit rates among patients who did not use medication and received psychosocial support initially were comparable to those who used a smoking cessation drug.

## INTRODUCTION

Tobacco use is the leading global health problem, causing more than 8 million deaths each year through debilitating diseases including cardiovascular and respiratory system diseases and more than 20 types of cancers of different sites^1^. Despite tobacco’s proven life threatening consequences, more than 1.3 billion people worldwide are still using tobacco products, mostly in low- and middle-income countries. The main ingredient of tobacco responsible for dependence is nicotine, which causes a relapsing disorder with a complex biological mechanism, including psychosocial dysregulation^2^. Currently, therapeutic interventions for nicotine dependence include pharmacological (varenicline, bupropion, and nicotine replacement) and behavioral therapies^3^. Up to now, numerous studies have comparatively evaluated the efficiency of these therapies, and the quit rates were highest for varenicline, followed by bupropion and nicotine replacement therapy (NRT). Nevertheless, there may be several differences between studies regarding relapse rates, long-term cessation rates, etc.

Several previous studies have evaluated the outcomes of smoking cessation interventions conducted in different settings in Turkey, and the quit rates varied from 22% to 51%^4-6^. The differences between the studies are due to several factors like cessation clinics’ infrastructural characteristics such as providing psychosocial support, or regulatory measures like providing cessation pharmacotherapies. In Turkey, smoking cessation services are entirely free of charge, and the Ministry of Health periodically offers cessation medications at the clinics. Nevertheless, there is a lack of studies comparing the long-term success rates of these medications. To the best of our knowledge, only one study evaluated the longterm quit rates of cessation medications and reported that varenicline had significantly higher abstinence rates than bupropion after 52 weeks^7^. Based on this background, this study aimed to compare the long-term outcomes, relapse rates, and the factors determining the long-term abstinence after smoking cessation interventions in Turkey.

## METHODS

This study was conducted at the Smoking Cessation Clinic of the Umraniye Research and Training Hospital, Istanbul, Turkey. The records of patients admitted for treatment between 2010 and 2018 were retrospectively evaluated, and baseline demographic and clinical data were recorded. The present study was approved by the Ethics Committee of Umraniye Research and Training Hospital (Date: 21.01.2021 and No: B.10.1.TKH.4.34.H.GP.0.01/4) and informed consent from each participant was obtained.

### Inclusion criteria

The patients who applied to the smoking cessation outpatient clinic between 1 January 2010 and 31 December 2018 and were treated either with varenicline, bupropion or nicotine replacement for three months, or followed up regularly without any medication but given just advice and motivational interventions, were included in the study.

All of the patients were evaluated monthly at control visits for three months and by phone call at 6 and 12 months following the first visit. At the first three follow-up visits, the smoking cessation status was asked and verified by carbon monoxide (CO) level in exhaled air. The status of smoking cessation was recorded in hospital files.

### Exclusion criteria

Patients lacking detailed information about the smoking status, and who had not used the given medication regularly for three months and who refused to answer the questionnaire during the phone call contact, were excluded.

### Interventions

Interventions for smoking cessation included either medication (varenicline, bupropion, or nicotine replacement therapy – NRT) or psychosocial support. Psychosocial support was given according to the guidelines of the Ministry of Health for smoking cessation, and included brief clinical interviews via the 5A (Ask, Advise, Assess, Assist, and Arrange follow-up) and motivating behavioral changes to quit via the 5R (Relevance, Risks, Rewards, Roadblocks, and Repetition) methods^8^. General tobacco education and self-help materials were given to all patients in the first visit. Besides psychosocial smoking cessation interventions including behavioral therapeutic approaches, motivational enhancement was performed at all visits. Telephone support was given monthly for all patients during the first 3 months and at the 6th month; also the current status of smoking was asked and recorded.

Short-term quit was defined as abstinence for 12 months. Long-term quit was defined as abstinence following first intervention until the time of the study.

### Definitions of the patients concerning smoking status

#### Long-term quitters

Patients who initially quit and maintained abstinence were classified as successful long-term quitters.

#### Relapsers

Patients who initially quit but resumed after a while were defined as relapsers.

#### Failed to quit

Patients who did not quit following the smoking cessation clinic visits and still smoking.

#### Late quitters

Patients who initially failed to quit with interventions in smoking cessation clinic, in first 6 months but quit independently after some period from the initial intervention.

In March 2019, 1254 patients were called by the smoking cessation program and certified by physicians, nurse, and medical secretary. Of these patients, 579 patients responded to the questions about current smoking situation and relapse. Demographic data, smoking status in first 12 months and medical history were extracted from hospital files. Low education level was defined as no education or primary or secondary education. High education level was defined as high school education and holding a university degree. Working status was dichotomized into active (those who had an occupation), and non-active (retired people, students, homemakers, and those unemployed).

### Statistical analysis

Descriptive statistics were presented as mean and standard deviation, or frequency and percentage, for continuous and categorical data, respectively. Testing of variables for normality was examined using visual (histogram and probability graphs) and analytical methods (Kolmogrov-Simirnov/Shapiro-Wilk tests). The demographic and clinical characteristics of the individuals, who currently smoked or quit at the time of the phone calls, were compared. The comparisons between independent study groups were made using the Mann-Whitney U and chi-squared tests, respectively. Univariate comparisons were used to determine the demographic and clinical variables, which are the candidates for multivariate analyses to identify the independent predictors of long-term abstinence. A p≤0.20 was considered the threshold in the univariate comparisons for the relevant parameter to be included in the multivariate analyses. The multivariate analyses were conducted using logistic regression analysis, and the model fit was evaluated using the Hosmer-Lemeshow test. A p<0.05 was considered to be statistically significant in univariate and multivariate analyses, which were conducted using PASW 18 (IBM Inc., Armonk, NY) software.

## RESULTS

### General characteristics

**Figure 1:**
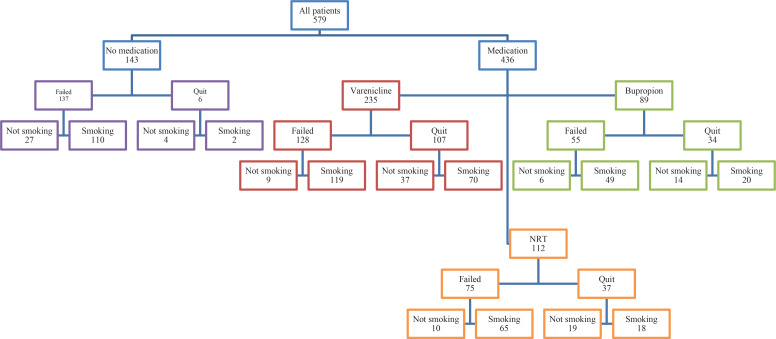
Flowchart of the study based on outcomes, Umraniye Training and Research Hospital, Istanbul, Turkey, 2020 (N=579)

A total of 579 patients (341 males and 238 females) with the mean age 50±12 years (range: 19–79) were included in the analyses. The median time from the date of visit to the smoking cessation clinic to analysis was five years (range: 2–10). The pharmacotherapies, including varenicline, bupropion, and NRT, were prescribed to 436 patients, and 143 patients were not found to be suitable for medication and were recommended only psychosocial support. The overall quit rate was 40.8% (n=236) in short- and long-term together; 31.8% (n=184) initially quit by the intervention, and 9% (n=52) quit some period after the intervention. The relapse rate after initial cessation was 19% (n=110) (Figure 1).

The proportion of patients who did not smoke at the time of analysis was 21.8% (n=126) and for those who were still smoking was 59.2% (n=343). Of these patients, 12.8% (n=74) maintained abstinence after the initial quit with intervention, and 9% (n=52) did not initially quit but gave-up smoking sometime after admission to the smoking cessation clinic.

### Univariate analysis

The comparisons between active smokers and quitters revealed that quit rates were higher among males (p<0.001), patients with a high level of education (p=0.003), those working in an occupation (p=0.043), and those who were living without a smoker at home (p=0.005). These parameters were also compared between individuals who initially quit and sustained abstinence (n=74) and those who relapsed after the initial quit (n=110), but no significant difference was found between the groups (Table 1).

**Table 1:** General characteristics of quitters and smokers, Umraniye Training and Research Hospital, Istanbul, Turkey, 2020 (N=579)

*Characteristics*	Long-term outcomes**			Short-term outcomes*		
	*Quitters (n=126) n (%)*	*Smokers (n=453) n (%)*	*p*	*Quit and sustained (n=74) n (%)*	*Quit but relapsed (n=110) n (%)*	*p*
**Sex**			<0.001			0.343
Male	92 (73.0)	249 (55.0)		52 (70.3)	69 (62.7)	
Female	34 (27.0)	204 (45.0)		22 (29.7)	41 (37.3)	
**Age** (years), mean±SD	51±14	50±11	0.342	51±15	50±11	0.683
**Education level**			0.003			0.293
Low	44 (36.4)	226 (51.7)		29 (41.4)	52 (49.5)	
High	77 (63.6)	211 (48.3)		41 (58.6)	53 (50.5)	
**Working status**			0.043			0.881
Active	60 (50.0)	171 (39.7)		36 (52.2)	56 (53.3)	
Non-active	60 (50.0)	260 (60.3)		33 (47.8)	49 (46.7)	
**FTND** score, mean±SD	5.67±2.33	6.15±2.14	0.041	5.86±2.33	6.30±1.94	0.193
**Other smoker(s) at home**			0.005			0.386
None	70 (69.3)	208 (53.7)		38 (66.7)	53 (58.9)	
Present	31 (30.7)	179 (46.3)		19 (33.3)	37 (41.1)	
**Comorbid disease**			0.701			0.601
None	80 (66.1)	297 (68.0)		50 (71.4)	80 (75.5)	
Present	41 (33.9)	140 (32.0)		20 (28.6)	26 (24.5)	
**Time since admission to smoking cessation clinic** (years)			0.401			0.793
<3	10 (8.0)	23 (5.1)		7 (9.5)	11 (10.0)	
3–7	52 (41.6)	206 (45.7)		33 (44.6)	54 (49.1)	
>7	63 (50.4)	222 (49.2)		34 (45.9)	45 (40.9)	
**Smoking cessation intervention**			0.613			0.17
Varenicline	46 (36.5)	189 (41.7)		37 (50.0)	70 (63.6)	
Bupropion	20 (15.9)	69 (15.2)		14 (18.9)	20 (18.2)	
NRT	29 (23.0)	83 (18.3)		19 (25.7)	18 (16.4)	
Psychosocial support	31 (24.6)	112 (24.7)		4 (5.4)	2 (1.8)	
**Quitting after intervention**			<0.001			
Failed to quit	52 (41.3)	343 (75.7)				
Quit successfully	74 (58.7)	110 (24.3)				
**Pharmacotherapy provision**			0.842			0.87
Free of charge	44 (55.0)	144 (53.7)		36 (57.1)	57 (59.4)	
Prescription	36 (45.0)	124 (46.3)		27 (42.9)	39 (40.6)	

FTND: Fagerström test for nicotine dependence. NRT: nicotine replacement therapy.

* Short term outcome: Abstinence or relapse status at month 12 following the first visit.

** Long term outcome: Abstinence or relapse status at the time of the study (2–10 years).

### Predictors of relapse

From the 184 patients who quit initially by the intervention at the smoking cessation clinic, 110 (59.8%) experienced a relapse and reinitiated smoking. Among these cases, 63.6% used varenicline, 18.2% used bupropion, 16.4% used NRT, and 1.8% received psychosocial support. The major causes of relapse were psychosocial problems, including stress and anxiety (n=49; 45%) and the urge to smoke (n=44; 40%) (Table 2). The distribution of causes of relapse was not significantly different between medication groups, including varenicline, bupropion, and NRT (p=0.075).

**Table 2:** Major factors responsible for relapse after initial successful quit, Umraniye Training and Research Hospital, Istanbul, Turkey, 2020 (N=110)

	*All patients*	*Varenicline*	*Bupropion*	*NRT*	*Psychosocial support*	*p ^a^*
	*n (%)*	*n (%)*	*n (%)*	*n (%)*	*n (%)*	
Psychological problems like stress and anxiety	49 (44.5)	35 (50.0)	9 (45.0)	3 (16.7)	2 (100)	0.075
Urge to smoking	44 (40.0)	24 (34.4)	8 (40.0)	12 (66.7)	0 (0)	
Lifestyle and social factors	7 (6.4)	6 (8.6)	1 (5.0)	0 (0)	0 (0)	
Weight gain	4 (3.7)	2 (2.8)	0 (0)	2 (11.1)	0 (0)	
Familial factors	3 (2.7)	2 (2.8)	1 (5.0)	0 (0)	0 (0)	
No answer	3 (2.7)	1 (1.4)	1 (5.0)	1 (5.5)	0 (0)	
Total	110 (100)	70 (100)	20 (100)	18 (100)	2 (100)	

a Comparison included only medication groups by excluding psychosocial support group.

NRT: nicotine replacement therapy.

### Relation of abstinence and medical treatment

The longest period of abstinence was in patients who used NRT (14.0±17.0 months), followed by those who used varenicline (9.5±12.7 months) and bupropion (8.2±14.8 months). The overall difference between the groups was statistically significant (p=0.048), and the *post hoc* pairwise comparisons revealed that this significance was due to the difference between NRT and varenicline (*post hoc* pairwise comparisons were as follows: Varenicline-Bupropion: p=0.888; Varenicline-NRT: p=0.039; NRT-Bupropion: p=0.043).

### Long-term results

The long-term outcomes according to the intervention are presented in Table 3. The overall long-term quit rates were similar between pharmacotherapy groups (p=0.103). However, when all groups were compared, the distribution of long-term outcomes was significantly different between intervention groups, including psychosocial support (p<0.001). Accordingly, the relapse rate was highest for varenicline, and the rate of unsuccessful quit attempts followed by a self-motivated and successful quit and failure to quit was highest for psychosocial support.

**Table 3:** Smoking status at the time of analyses, Umraniye Training and Research Hospital, Istanbul, Turkey, 2020 (N=579)

	*Varenicline*	*Bupropion*	*NRT*	*Psychosocial support*	*p ^a^*
	*n (%)*	*n (%)*	*n (%)*	*n (%)*	
Long-term successful quit	37 (15.7)	14 (15.7)	19 (17.0)	4 (2.8)	<0.001
Relapse after initial quit	70 (29.8)	20 (22.5)	18 (16.1)	2 (1.4)	
Quit after a period from initial intervention	9 (3.8)	6 (6.7)	10 (8.9)	27 (18.9)	
Fail to quit	119 (50.6)	49 (55.1)	65 (58.0)	110 (76.9)	

a p<0.001 when all intervention groups were compared; p=0.103 when only medications (varenicline, bupropion, NRT) were compared.

NRT: nicotine replacement therapy.

The determinants of successful long-term abstinence were evaluated by a logistic regression model (Table 4). The analysis of candidate parameters (sex, Fagerström Nicotine Test for Nicotine Dependence score^9^, having another smoker at home, quitting by intervention) from the univariate analyses revealed that male sex (OR=2.3; 95% CI: 1.4–4.0, p=0.002), having no other smoker at home (OR=1.7; 95% CI: 1.01–2.8, p=0.044), and initial successful quit by the intervention (OR=4.1; 95% CI: 2.6–6.6, p<0.001) were the significant predictors of complete long-term abstinence.

**Table 4:** Multivariate analysis of determinants of successful abstinence, Umraniye Training and Research Hospital, Istanbul, Turkey, 2020 (N=126)

*Variable*	*OR*	*95% CI*	*p*
Sex (male)	2.3	1.4–4.0	0.002
Having no other smoker at home	1.7	1.01–2.8	0.044
Quitting by intervention	4.1	2.6–6.6	<0.001

FTND: Fagerström test for nicotine dependence. CI: confidence interval. OR: odds ratio. Gender, FTND score, having another smoker at home, quitting by intervention were included in the model. FTND score was not statistically associated.

## DISCUSSION

Based on the results of the present study, the overall success rate of our smoking cessation program was 21.8%; the highest overall success rate was found in the NRT group, but there was only a modest difference with other drugs. This finding was interesting because the current evidence in the literature suggests that varenicline is superior to NRT^10^, and it is recommended as the first-line treatment among smoking cessation pharmacotherapies^11^. Nevertheless, this should be interpreted cautiously since multiple exogenous factors may confound the cessation status. Many studies from Turkey reported the quit rates in various smoking cessation clinics. Esmer et al.^4^ reported the rate of abstinence for at least three months as 53%, and for more than 36 months (longterm outcome) as 22%. In another study, Pekel et al.^5^ reported the 1-year quit rate and relapse rate as 30.1% and 50.1%, respectively. Another study by Durmus et al.6 reported the 1-year quit rate as 24% and relapse rate as 51.4%. The quit rates in these studies are generally similar to international examples and vary between 20% and 50%. The wide range of distribution is mainly related to the mode of delivery of cessation support in different centers. Accompanying psychosocial support, providing medications, individual counseling and group therapy opportunities, the number of follow-up visits, either onsite or by phone all, play a role in the clinics’ overall success rate.

In the study, the biochemical confirmation of adherence to pharmacotherapy and abstinence could not be measured except for the first three visits performed via the measurement of exhaled CO level, but the data for use of the given medication and quitting after the third month depended on self-reported non-smoking. The golden standard for evaluating quitting status includes measurement of the presence of smoking-related substances in exhaled breath, saliva, urine or blood. Still, the biochemical tests may not reflect the smoking status accurately for irregular smokers.

Smoking cessation is the most significant health intervention for promoting health^12^. It is also one of the most critical public health interventions worldwide, regardless of the method to quit smoking successfully. The World Health Organization conceptualized the essential points for reducing the demand for tobacco products in six key targets, also called MPOWER strategies^13^. Turkey has been the first country to fully implement the MPOWER package in the world^14^. The ‘O’ in the MPOWER stands for offering smokers help to quit tobacco use, which is a multidimensional topic including quitlines, outpatient services, medications, counseling, and similar supports. Except for the medications, these services are delivered to the general public free of charge in Turkey. The social insurance system does not reimburse the medications (varenicline, bupropion, NRT), and patients can buy them upon prescription. The Ministry of Health periodically offers these medications free of charge in smoking cessation clinics if an individual applies for smoking cessation and is found to be eligible for medicine-based intervention after a medical assessment by a certified physician in smoking cessation responsible for the treatment. These services are provided since 2010, but the comparative effectiveness of medications has been evaluated only in a few studies.

The present study revealed that providing the medications free of charge to patients had no significant effect on quit rates. Although the number of reports about this topic is limited, it is a critical outcome since providing medications was a significant motivator for smoking cessation in previous studies^15,16^. The main reason for this assumption was that the financial burden of cessation medications was regarded as a barrier for smokers who seek professional support to quit^17^. Nevertheless, we found that supplying the drugs free of charge did not affect long-term quit success. This is consistent with other studies from Turkey. Salepci et al.^18^ and Karadoğan et al.^19^ compared the outcomes of patients who used paid treatment and free medications, and reported no difference in short-term and long-term quit rates between the groups, but providing free drugs increases the number of smokers who try to quit. In those studies, the only significant factor associated with quitting was the patient age, which showed that quit rates increased as the age increases, which was not found in our study. Our results showed that male sex, having no other smoker in the same house, and initial success due to intervention in smoking cessation clinics were the independent predictors of a successful quit.

Another striking outcome was that a successful quit after initial failure was highest in the psychosocial support group. It is known that about seven of ten current smokers have the will to quit^2^, and even simple motivational psychosocial support such as brief advice from a physician can increase the odds of quitting by 70%^11^. One can assume this high rate of abstinence after initial failure may be associated with the steps of behavioral change. In the study by Siewchaisakul et al.^20^, healthcare professionals’ direct advice about quitting was shown to facilitate the transition from contemplation and preparation phases of the behavioral change. This might also be the case for our patients, who experienced a behavioral transition towards cessation after visiting our clinic even without receiving medication for quitting. Nevertheless, the possible effects of other confounders have to be considered when commenting on the high quit rates in patients who did not take a pharmacotherapeutic agent for quitting. A previous study by Kocak and Akturk^21^ reported that providing no pharmacotherapy was associated with decreased adherence to smoking cessation, and supplying medicine increased the commitment to cessation five times higher (OR=5.0; 95% CI: 2.8–8.9). Keeping this in mind, we believe that admission to a smoking cessation clinic is one of the main motivators of quitting and may have a longterm effect on increased awareness towards quitting.

### Strengths and limitations

The smoking quitting status of the patients was examined and evaluated in detail, including the relapse time, initial or late term quit following the interview in smoking cessation clinic, and long-term data about abstinence were strengths of the study. But there were also a few limitations. First, the shortterm quitting was evaluated with measurement of CO in exhaled air for the first three months but longterm abstinence was recorded according to patients’ self-report, which may include incorrect data. Second, the information about adherence to given medications was also recorded according to patients’ self-report. Third, detailed data concerning the adverse effects and abstinence symptoms were lacking.

## CONCLUSIONS

In our cases, the overall long-term quit rate was 21.8%. The independent predictors of successful abstinence were male sex, having no other smoker in the same house, and initial successful quit by intervention at cessation clinic. Providing cessation medications free of charge did not affect the long-term outcomes.

## Data Availability

The data supporting this research is available from the authors on reasonable request.
